# Novel carbon film induces precocious calcium oscillation to promote neuronal cell maturation

**DOI:** 10.1038/s41598-020-74535-6

**Published:** 2020-10-19

**Authors:** Anastasia Ludwig, Sebnem Kesaf, Joonas J. Heikkinen, Tatiana Sukhanova, Shokoufeh Khakipoor, Florence Molinari, Christophe Pellegrino, Sung I. Kim, Jeon G. Han, Henri J. Huttunen, Sari E. Lauri, Sami Franssila, Ville Jokinen, Claudio Rivera

**Affiliations:** 1 HiLIFE, Neuroscience Center, University of Helsinki, Haartmaninkatu 8, 00290 Helsinki, Finland; 2 Aalto University, Micronova, Tietotie 3, 02150 Espoo, Finland; 3 Sungkyunkwan University, Suwon, South Korea; 4 INMED (INSERM U1249), Aix-Marseille Université, Marseille, France; 5 MMG, INSERM, Aix-Marseille Université, Marseille, France; 6 Present Address: Hertie Institute for Clinical Brain Research, University of Tübingen, 72076 Tübingen, Germany

**Keywords:** Regeneration and repair in the nervous system, Nanoscale materials

## Abstract

Different types of carbon materials are biocompatible with neural cells and can promote maturation. The mechanism of this effect is not clear. Here we have tested the capacity of a carbon material composed of amorphous sp^3^ carbon backbone, embedded with a percolating network of sp^2^ carbon domains to sustain neuronal cultures. We found that cortical neurons survive and develop faster on this novel carbon material. After 3 days in culture, there is a precocious increase in the frequency of neuronal activity and in the expression of maturation marker KCC2 on carbon films as compared to a commonly used glass surface. Accelerated development is accompanied by a dramatic increase in neuronal dendrite arborization. The mechanism for the precocious maturation involves the activation of intracellular calcium oscillations by the carbon material already after 1 day in culture. Carbon-induced oscillations are independent of network activity and reflect intrinsic spontaneous activation of developing neurons. Thus, these results reveal a novel mechanism for carbon material-induced neuronal survival and maturation.

## Introduction

Brain trauma as well as neurodegenerative diseases are the leading cause of irreversible disability and low quality of life in the elderly population^1^. A way to combat neurodegeneration is to promote reparation of neuronal networks, rewiring of neuronal connections, and eventual restoration or substitution of the lost functionality^2^. One putative therapeutic avenue is providing scaffolds—special materials that support targeted differentiation of neuronal stem cells and neurite outgrowth of regenerating neurons. Carbon-derived materials possess numerous properties that make them usable as scaffolds^3^. Carbon nanotubes (CNTs) and graphene are among the most studied carbon materials for biological applications. These materials enhance neuronal stem cell differentiation^4–6^, as well as promote neuronal survival, neuronal activity, and neuronal process outgrowth^7–13^. Neurons cultured on CNTs have increased levels of neuronal K^+^–Cl^−^ cotransporter KCC2, a key component in the functional maturation of inhibitory synaptic^14^ and glutamatergic^15–17^ transmission. Downregulation of this protein is also implicated in reactive plasticity following brain trauma^18^. CNTs have been suggested to improve the electrical responsiveness of neurons by facilitating local electronic shortcuts between somas and dendrites^19^. The ability of CNTs to form tight contacts with neurons is beneficial for neuron-electrode interfaces^20–24^. 3D graphene substrates support growth and differentiation of neurons^25–27^ that in combination with anti-inflammatory properties^28,29^ makes graphene a next-generation neuronal tissue scaffold.

Despite the importance of novel carbon materials for future engineering, we do not fully understand the mechanisms underlining the trophic action of carbon scaffolds. In this work, we propose a novel mechanism by which a new type of sputtered carbon material accelerates neuronal maturation. The carbon film material consists of conducting nanoscale sp^2^ carbon islands embedded in a diamond-like sp^3^ carbon matrix. We demonstrate that dissociated hippocampal neurons grown on the carbon films show increased arborisation and upregulated expression of KCC2 as compared to a glass-cultured control. Importantly, we found that the carbon material induces an early increase in low frequency spontaneous intracellular calcium oscillations that are independent of the network activity. These oscillations are primarily generated by intracellular mechanisms and are in the range known to stimulate the activation and expression of proteins involved in cell maturation^30,31^. Thus, apart from demonstrating the suitability of the novel material for neuronal interface, we also provide new insight into the mechanism for its neurotrophic action.

## Materials and methods

### Carbon deposition and characterization

**Figure 1:**
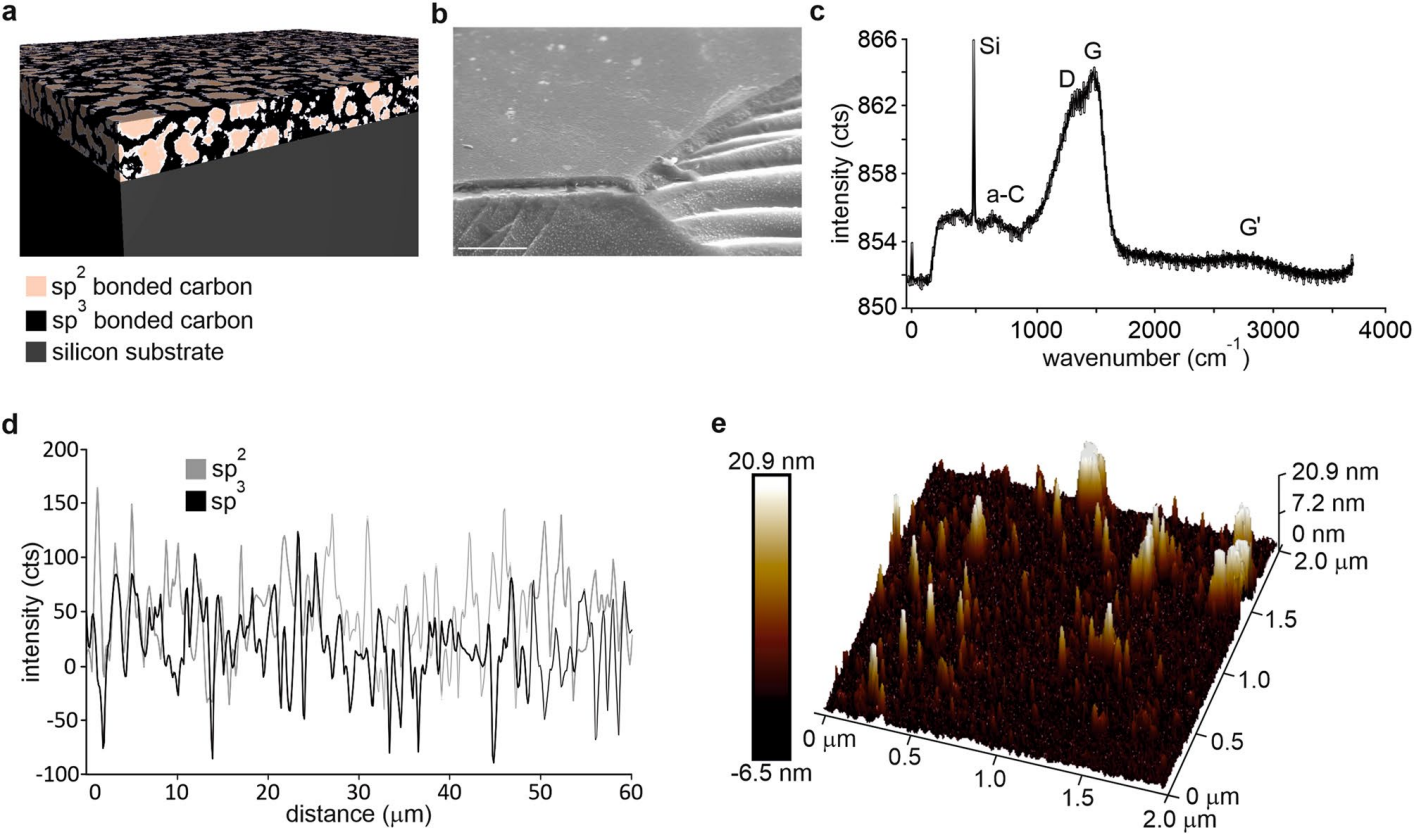
Structure of nC films: (**a**) schematic showing the sp^2^ and sp^3^ domains; (**b**) scanning electron micrograph picture showing the carbon thin film on top of silicon substrate, scale bar 1 μm; (**c**) micro Raman spectrum showing characteristic peaks; (**d**) line scan of 50 µm showing spatial distribution of G and D peaks; (**e**) AFM surface scan over 4 µm^2^ area.

The carbon material studied (hereafter referred to as nanocarbon, nC) consists of nanoscale sp^2^ carbon islands embedded in sp^3^ carbon matrix (Fig. 1a). nC was deposited with close field unbalanced magnetron sputtering with two graphite targets on top of a silicon wafer as reported in a previous publication^32^. The thickness of the nC films was 500 nm.

As a reference carbon material, we utilized tetrahedral amorphous carbon (ta-C), a type of diamond-like carbon with high sp^3^ content, deposited on top of a silicon wafer. The thickness of the ta-C film was 30 nm with a resistivity of ≈ 10^4^ Ωcm and roughness of around 1 nm. The deposition of ta-C is explained in an earlier publication^33^**.** In order to study the effect of resistivity, we also used conductive indium tin oxide (ITO) slides (purchased from Mersk, resistivity 8–12 Ω/sq) as an additional reference.

For scanning electron micrographs, SEM Zeiss Supra 40 was used. Raman spectrum was obtained with WITec Alpha 300 RA with scanning confocal Raman spectroscopy. Parameters for the measurement were: 50 µm scan length, 50 measurement points in line, 10 accumulations per point, 0.5 s integration time for one scan. The peaks were fitted with Lorenz equations, and the peak maximum height was used to calculate average I_D_/I_G_ peak ratio. For surface roughness data, atomic force microscopy (AFM), Veeco Dimension 5000 was used. Resistivity measurement was done with four-point probe Loresta AP. One 100 mm wafer was scanned from eight locations to acquire averaged data.

### Cell cultures

Dissociated hippocampal cultures were prepared from embryonic day 17 (E17) rats as described elsewhere^34–36^ and seeded on poly-l-lysine treated nanocarbon films or glass coverslips at 105 cells/cm^2^ in Neurobasal medium containing B27 supplement and 0.5 mM l-glutamine (Gibco/Life Technologies). The permissive area for cell growth was restricted to a similar size in all experiments. Before plating, the medium was pre-incubated on astroglial culture for 24 h. Neuronal cultures were fed once a week by changing half of the medium. Astroglial cultures were prepared according to Banker and Goslin^34^. All animal experiments were approved by ELLA—The National Animal Experiment Board of Finland. The experiments were performed in accordance with the relevant guidelines and regulations of the University of Helsinki. Timed-pregnant mice were euthanized by CO_2_, and cervical dislocation. The day of vaginal plug was defined as embryonic day 0.5 (E0.5).

### Transfection of neurons and Sholl analysis

To visualize neurons for Sholl analysis, DIV (day in vitro) 6 cultures grown on nanocarbon and glass substrates were transfected using Lipofectamine (LF) 2000 protocol (Life Technologies) with a DNA construct encoding fluorescent protein mCherry. Culture medium was replaced with plain Neurobasal without any supplements 20–30 min before transfection. The original culture media from the well was saved for the duration of the transfection (4–5 h). LF (2 µl) and DNA (1 µg) were separately diluted in 50 µl of Neurobasal media and kept at room temperature for 5 min, then LF solution was added to DNA solution, mixed, and incubated 20 min. DNA/LF mixture was added to cultured neurons and incubated on cells (37 °C, 5% CO_2_, 95% air) during 4–5 h. After the incubation, the transfection media was replaced with the original culture media.

**Figure 2:**
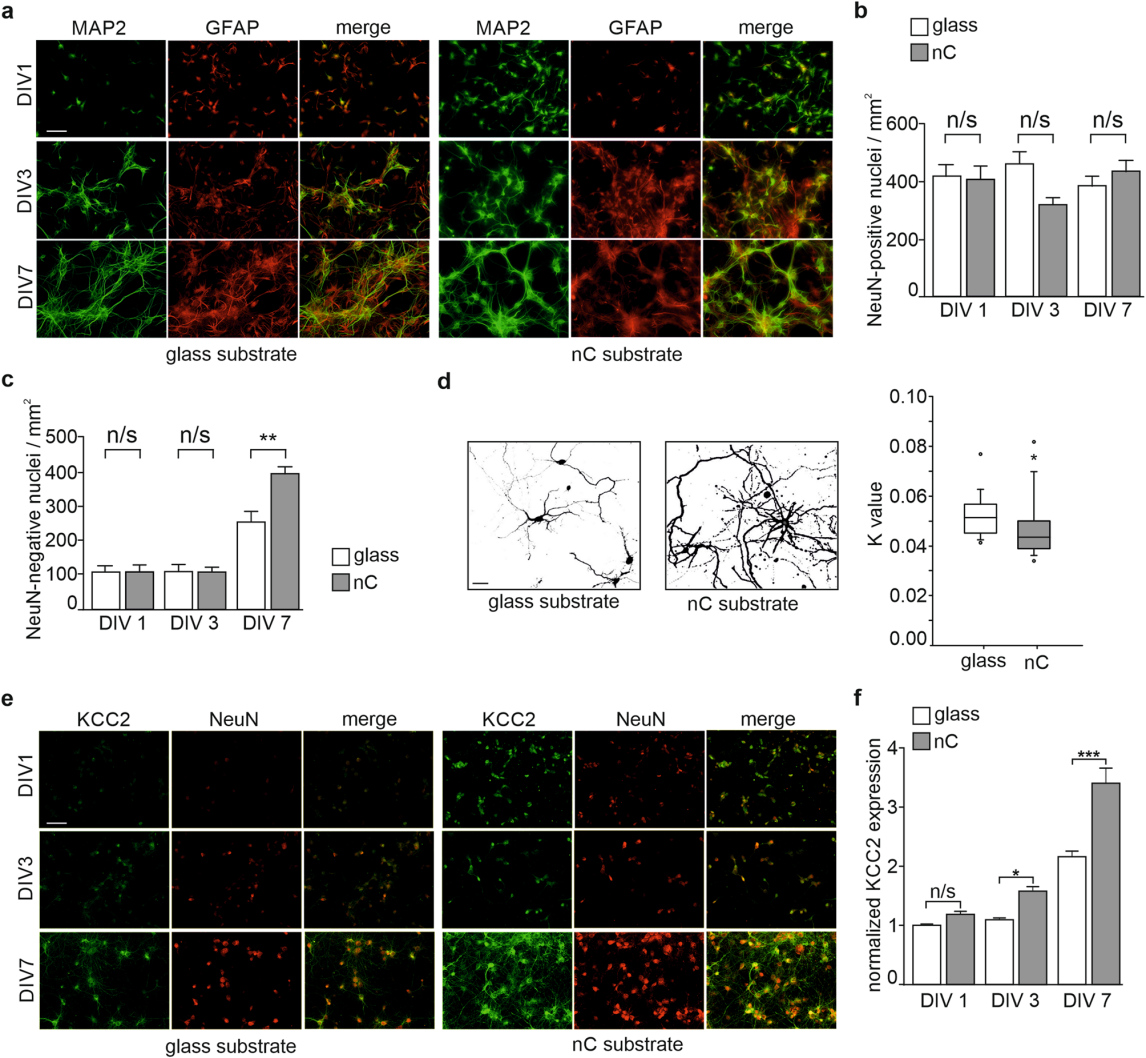
Growth of hippocampal neurons and expression of KCC2 on glass or nC substrates: (**a**) anti-MAP2 (green) and anti-GFAP (red) immunostaining, scale bar 50 μm; (**b**) number of NeuN-positive cells in cultures grown on glass or nC substrates (mean values, error bars represent SEM; 3 independent cultures, n = 35 glass and 41 nC fields of view); (**c**) number of NeuN-negative cells (mean values, error bars represent SEM; 3 independent cultures, n = 35 glass and 41 nC fields of view); (**d**) morphology of cultured neurons at DIV 7 (scale bar 20 μm) and Sholl analysis of dendritic arborisation, K—regression coefficient corresponding to the rate of dendritic arborization decay over the distance from neuron soma (*P = 0.045, Mann–Whitney test n = 21 glass and 25 nC); (**e**) anti-KCC2 (green) and anti-NeuN (red) immunostaining, scale bar 50 μm; (**f**) quantification of KCC2 expression in neurons grown on glass or nC substrates (mean values, error bars represent SEM; 3 independent cultures, n = 34 control and 39 carbon fields of view).

Neurons expressing mCherry were imaged 24 h after transfection. Neuronal arborization was calculated using Scholl analysis (ImageJ/Fiji software). This test creates a series of concentric shells around the soma of the cell and counts how many times arbors intersects the shells. The transfection efficiency of the cultures was adjusted so that the arborization could be analyzed in high-density cultures. Analysis was performed using semi-log measurement, which calculated log of intersection per area over distance. Rates of decay (K value, regression coefficient of slope) of dendritic arborization were calculated based on the slope of semi-log measurements^37^. The lower rate of decay is an indication of higher dendritic arborization around soma (Fig. 2d, right panel).

### Immunostaining and image analysis

Cultures were fixed with 4% paraformaldehyde in phosphate-buffered saline (PBS) at room temperature and permeabilized with 0.1% Triton X-100 in PBS. Cells were blocked with 10% donkey serum, 0.1% Triton X-100 in PBS at room temperature and then incubated in primary antibody (2% donkey serum, 0.1% Triton X-100 in PBS) at 4º C overnight. The following antibodies were used: mouse anti-MAP2 at 1:500 (Chemicon), rabbit anti-GFAP (1:400 Chemicon), and mouse anti-NeuN at 1:400 (Milipore). Next day samples were incubated with secondary antibodies (anti-rabbit Alexa Fluor 488 and anti-mouse Alexa Fluor 568, both from Molecular Probes at 1:400 dilution) for 1 h, stained cell nuclei with Hoechst (Molecular Probes, 1:1000) washed and mounted in Prolong Gold mounting media (Life Technologies). Fluorescent wide-field images were acquired with Zeiss AxioImager.M1 microscope equipped with AxioCam HR camera and 40× objective ECPlan-Nerofluar/0.75/Ph2. For image analysis, we used custom-made plugin for ImageJ software. For analysis presented in Fig. 2b we quantified the number of nuclei positive for both NeuN and Hoechst immunoreactivity. For data shown in Fig. 2f we measured average intensity of KCC2 immunoreactivity in NeuN-positive cell bodies.

### Calcium imaging

For the imaging of intracellular calcium fluctuations cells were loaded with cell-permeable fluorescent calcium indicator Fluo-4 AM (Life Technologies). Fluo-4 AM was diluted in culture medium to final concentration of 0.04 μM. After 30 min incubation with the dye, coverslips and nanocarbon films with neurons were transferred to an imaging chamber filled with 1 ml HEPES-buffered physiological salt solution containing (mM): NaCl (127), KCl (3), MgCl_2_*6H_2_O (1.3), CaCl_2_*2H_2_O (2), glucose (10), and HEPES (20) (pH 7.4) and imaged at 37 °C using Zeiss LSM 710/Axio confocal microscope controlled by ZEN 2011 software and equipped with water immersion objective (Zeiss 20×/1.0).

Fluorescence of calcium indicator Fluo-4 was excited with Argon Laser (Lasos Lasertechnik GmbH) at 488 nm and emission was collected at 520–620 nm. The pinhole was fully opened. Scanning was performed in XY mode during 1800s, at 0.5 s per frame, with resolution of 0.83 × 0.83 μm/pixel resulting in a recording field of 425 × 425 μm. The following blockers of neuronal activity were added to the recording medium in some experiments as indicated in the text: a blocker of voltage-gated sodium channels tetrodotoxin (TTX, 1 μM), antagonist of AMPA receptors 6-cyano-7-nitroquinoxaline-2,3-dione (CNQX, 10 μM), antagonist of NMDA receptors (2R)-amino-5-phosphonopentanoate (APV, 50 μM), antagonist of GABA_A_ receptors bicuculline (BMI 20 μM), as well as blockers of L-type voltage-gated calcium channels CdCl2 and NiCl2 (100 μM each).

## Results and discussion

The nanocarbon films utilized in this study were fabricated by close field unbalanced magnetron sputtering as reported previously^32^. The films were deposited on top of silicon substrates (<100>, p-type doping, 30–50 Ωcm, 500 µm) with nC layer thickness of 500 nm. Figure 1a displays a schematic illustration of the material structure with nanoscale sp^2^ bonded carbon islands in a sp^3^ matrix. Figure 1b shows a scanning electron micrograph of the nC film on top of a silicon chip. The composition of nC was characterized by micro Raman spectroscopy (Fig. 1c). The graph shown in Fig. 1c is an averaged graph from 500 measurements. Two characteristic peaks for carbonaceous materials were clearly visible in the data: the D-peak at 1360 cm^−1^ and G-peak at 1560 cm^−1^, which correspond to the disordered peak (sp^2^ chains) and the graphitic peak (sp^2^ rings) respectively^17^. The sp^3^ content can be estimated with the I_D_/I_G_ ratio. The I_D_/I_G_ ratio was 0.85 and the G-peak was located around 1543 cm^−1^. This yields sp^3^ content to be around 15–20%. Spatial variation of sp^2^ rings and chains is shown in Fig. 1d. The nC film was characterized by AFM for surface roughness. Figure 1e shows a typical result of an AFM scan over a 2 µm × 2 µm area. The root mean square roughness was 2.7 nm and the projected surface area was 4.05 μm^2^, so the surface was found to be relatively smooth. The electrical properties of the nC film were characterized by a four-point probe measurement. The resistivity of the film was 3.2 Ωcm, which lies in-between carbon films rich in sp^3^ (> 10^12^ Ωcm) and sp^2^ graphitic crystals (10^–2^–10^–5^ Ωcm)^38^.

In order to assess if the nC films support development of neurons, we cultured dissociated hippocampal neurons on nC and compared them to neurons cultured on conventional glass substrate. For visualization, cultured cells were fixed at day in vitro (DIV) 1, 3, and 7 and processed for immunostaining. Anti-MAP2 (microtubule-associated protein 2; neuronal marker) and anti-GFAP (glial fibrillary acidic protein; astrocyte marker) antibodies were used to reveal neurons and glial cells. As expected, we found a gradual increase of the area covered by cultured cells during the 7 days of the experiment (Fig. 2a).

To analyze the density of cultured neurons, we quantified neuronal nuclei revealed by anti-NeuN (neuronal-specific nuclear protein) antibodies. There was no significant difference in neuronal cell density between cultures cultivated on the nC films or glass substrates at any of the analyzed developmental stages (Fig. 2b). Comparison of the number of NeuN negative non-picnotic cell nuclei between nC and glass substrates showed a significant increase at DIV 7 in nC. This most likely corresponds to an increase of glial cell population (Fig. 2c) in agreement with the ability of glia cells to undergo cell division, in contrast to differentiated neurons.

We quantified the complexity of the dendritic arborisation using Sholl analysis of GFP-expressing dendrites. Here, we found that at DIV 7 neurons cultivated on nC films had more branched and longer neurites than those grown on glass (Fig. 2d). Thus, nC substrate facilitated morphological maturation of neurons. Next, we studied whether nC films could also promote faster neuronal maturation. KCC2 is a well-documented marker of neuronal cell maturation, in particular of the maturation of GABAergic^39,40^ and glutamatergic^15,16^ neurotransmission. We performed immunostaining with anti-KCC2 and anti-NeuN antibodies (Fig. 2e) and quantified the intensity of KCC2 immunoreactivity in NeuN-positive neurons. Cultures grown on nC films showed significantly higher expression of KCC2 starting already at DIV 3 and the difference increased even further at DIV 7 (Fig. 2f).

A reference carbon substrate (ta-C) with limited conductivity and surface roughness had a clearly different effect on neuronal cultures and was less cell culture compatible. Most notably surviving neurons aggregated in clusters and displayed abnormal dendritic morphology (Fig. S1). The reference carbon material ta-C was similarly characterized by Raman. The I_D_/I_G_ ratio for this film was 0.15 and the location of the G-peak is around 1556 cm^−1^ that gives a sp^3^ content estimate of over 85%. Compared to the nC film, the ta-C is somewhat smoother and has four orders of magnitude higher resistivity due to the lower sp^2^ content.

**Figure 3:**
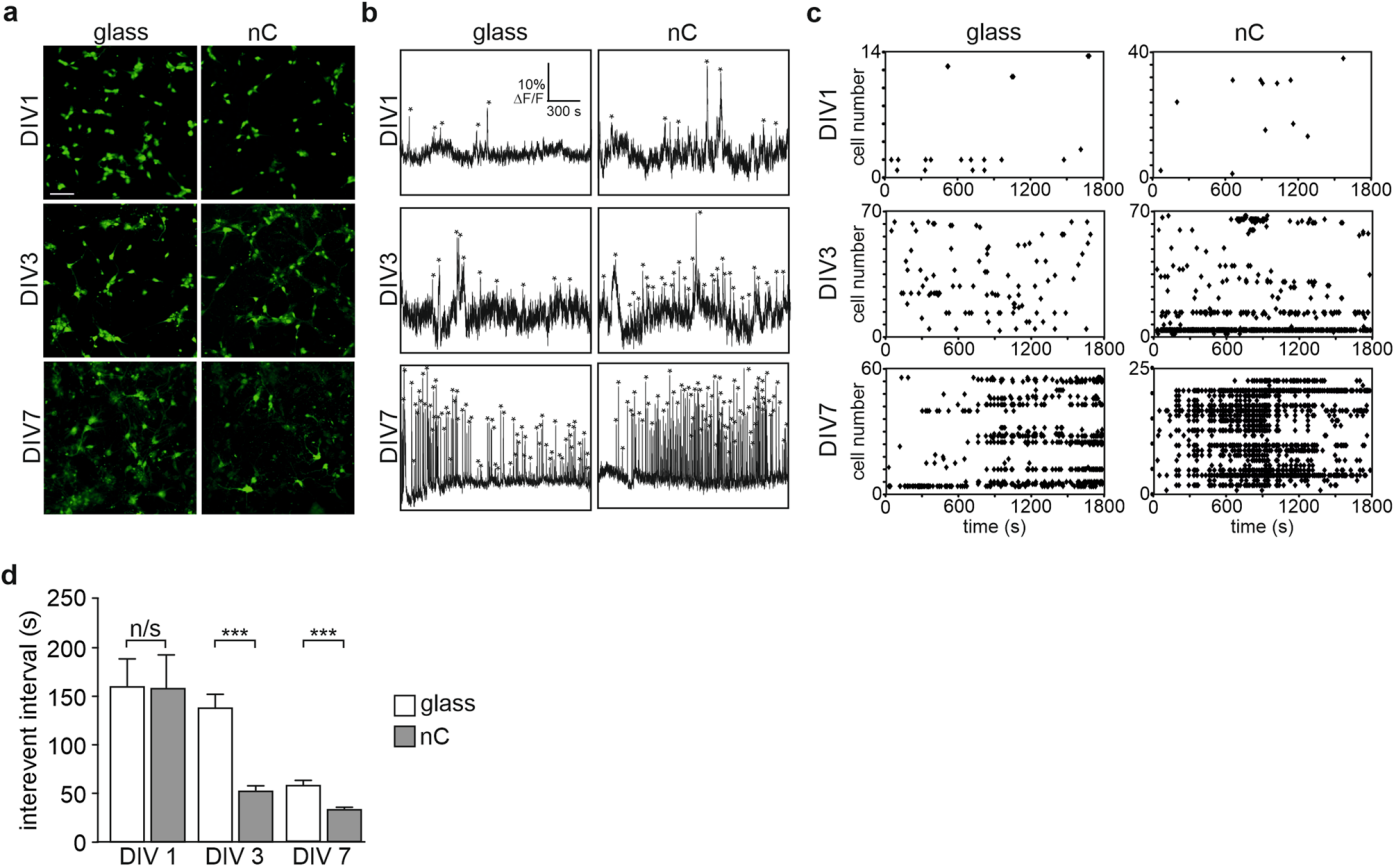
Recordings of intracellular calcium oscillations: (**a**) neuronal cultures loaded with calcium indicator Fluo-4, scale bar 50 μm; (**b**) representative traces of intracellular calcium fluctuations, activity peaks are marked with asterisks; (**c**) representative raster plots of the neuronal activity in the field, individual calcium peaks are marked as dots plotted vs time (X-axis) and vs cell serial number (Y-axis); (**d**) intervals between calcium peaks in neurons grown on nC or glass substrates (mean values, error bars represent SEM; 3 independent cultures; at DIV 1: glass n = 105, nC n = 31; at DIV 3 glass n = 208, nC n = 502; at DIV 7 glass n = 540, nC n = 1601 events, ***p < 0.0001, two-way ANOVA with Bonferroni post-hoc test).

The spatio-temporal pattern of intracellular calcium fluctuation plays a crucial role in neuronal development^30,31^. Therefore, we studied whether the nC film could impose a change in the developing intracellular calcium activity. Activity of cultured neurons was studied at DIV 1, 3 and 7. Cells were loaded with cell-permeable fluorescent calcium indicator Fluo-4 AM and calcium activity was recorded using confocal microscope at 0.5 s per frame. A recording field of 425 × 425 μm typically included 40–80 cells (Fig. 3a). The intensity of Fluo-4 is positively correlated with intracellular calcium concentration, and thus faithfully follows depolarization of neurons (Fig. 3b). At the end of each recording cultures were stimulated with 50 μM glutamate in order to determine the viability of neurons. Only cells that responded to the glutamate application with a fast and robust increase of intracellular calcium were taken in consideration for the analysis. An additional criterion for selection was the morphology and the size of the loaded cell. Since the cultures were plated from embryonic day 17 hippocampi, the glia content in the freshly plated cultures was extremely low (see, for example, Banker and Goslin^34^), thus the large majority of recorded cells were neurons. We analyzed the data with a custom-made software as previously described^41^. In order to determine activity events, fluorescent intensity of each neuron was normalized to the baseline level (∆F/F) and plotted as a function of time (Fig. 3b). Calcium peaks were identified as robust increases of ∆F/F above 100% of the noise level (marked with asterisks at Fig. 3b).

Maturation of neuronal networks is accompanied by an increase in the frequency of event activity, reflecting progressive synaptogenesis. This can be appreciated form raster plots where calcium peaks were marked as dots and plotted vs time (X-axis) and vs cell serial number (Y-axis) (Fig. 3c). Accordingly, the average interval between calcium peaks decreased with culture development (Fig. 3d). Interestingly, culturing on the nC substrate lead to significantly reduced interval between calcium peaks at DIV 3 and DIV 7 as compared to the glass substrate (53 ± 6 s. vs. 138 ± 13 s at DIV 3, Student t-test, p < 0.0001; and 34 ± 2 s. vs 59 ± 5 s. at DIV 7, Student t-test, p < 0.0001). In fact, neurons growing on nC films reached at DIV 3 the same frequency of calcium events as neurons cultured on glass coverslips reached only at DIV 7. Thus, the nC substrate promoted maturation of neurons at early stages. This effect was not transient as enhanced network activity was still significant at DIV 7. Interestingly, when neurons were grown on the ITO substrate, the developmental increase in activity was not significantly different from cultures grown on glass (Fig. S3a). Thus, the mechanism behind the effect produced by the nanocarbon substrate may not be entirely dependent on increased conductivity of the material.

**Figure 4:**
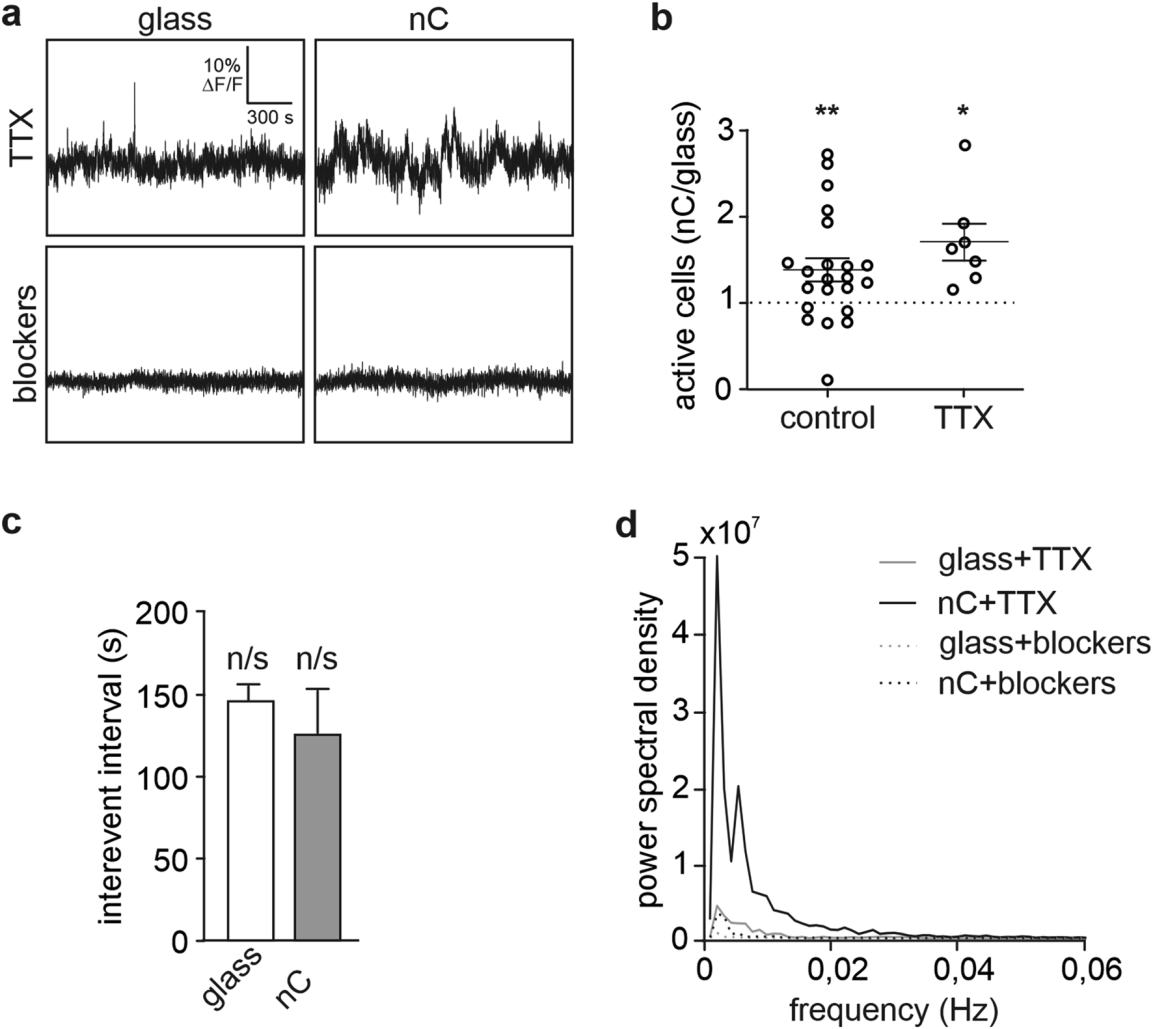
Intracellular calcium oscillations in DIV 1 neurons after application of activity blockers: (**a**) representative traces of intracellular calcium fluctuations in the presence of TTX or the blocker cocktail; (**b**) relative number of active cells in DIV 1 cultures grown on nC substrate, control vs. TTX (individual values are plotted as open circles, mean value is plotted as the horizontal line, error bars represent SEM), the number of active cells in sister cultures grown on glass is set to 1 and marked with the dashed line (control: 7 independent cultures, n = 21 individual coverslips; TTX: 3 independent cultures, n = 7 individual coverslips, *p = 0.018, **p = 0.0065, one-sample t-test); (**c**) intervals between calcium peaks in DIV 1 neurons grown on nC or glass substrates and recorded in the presence of TTX (mean values, error bars represent SEM; 3 independent cultures; glass n = 292, nC n = 59; n/s: p > 0.9 when compared to same-substrate cultures recorded without TTX (Fig. 3d), two-way ANOVA with Bonferroni post-hoc test); (**d**) mean power spectral density curves of neuronal activity frequencies before and after the blockers application (3 independent cultures; glass + TTX n = 47, nC + TTX n = 40, glass + blockers n = 14, nC + blockers n = 46 cells recorded).

Next, we aimed to find out if nanocarbon substrate promotes calcium oscillations already from the beginning of the culturing period. At DIV 1, neuronal activity is sparse and many cells remain silent for prolonged periods of time. Synaptogenesis and network-driven activity starts at later developmental stages, thus the mechanism driving calcium oscillations in DIV 1 cultures is mainly intrinsic and could be affected by the expression of ion-channels and neurotransmitter receptors in the plasma membrane. To estimate the level of activity at this early stage, we counted active cells, i.e. neurons that exhibited one or more calcium peak within the recording period. There were 1.4 times more active cells in cultures grown on nC film as compared to the sister cultures grown on glass (p = 0.009, one-sample t-test; Fig. 4b). The substrate-related difference in the number of active cells was present only at the very early stage of culture development and could not be detected later at DIV 3 and DIV 7 (Fig. S2).

To investigate further enhanced activity at DIV 1, calcium oscillations were recorded in the presence of TTX (1 μM, Fig. 4a). TTX is a blocker of voltage-gated sodium channels that inhibits generation of action potentials and network activity, thus isolating spontaneous activity of a single cell. At DIV 1 there was no effect of TTX on the number of active cells neither on glass, nor on nC films (glass control 40 ± 6% vs glass TTX 46 ± 12%, Student t-test, p = 0.62; nC control 55 ± 7% vs nC TTX 64 ± 9%, Student t-test, p = 0.50). Interestingly, when comparing the two substrates, the difference between glass and nanocarbon was preserved, i.e. in the presence of TTX, the culture on nC film had 1.7 times more active cells than sister cultures grown on glass (p = 0.018, one-sample t-test; Fig. 4b). Average interval between calcium peaks at DIV 1 was also not affected by the TTX treatment (Compare Fig. 3d (DIV 1) and Fig. 4c; glass control 160 ± 28 s. vs glass TTX 146 ± 10 s., Student t-test, p = 0.57; nC control 158 ± 34 s. vs nC TTX 126 ± 27 s., Student t-test, p = 0.48).

Analysis of the power spectral density of the traces revealed that the predominant frequencies of calcium oscillations in DIV 1 cultures were less than 0.02 Hz (Fig. 4d). This is in a good agreement with previously published data, showing that average frequency of calcium oscillations in immature neuronal cultures was one event per 60 s, i.e. 0.017 Hz^30,31^. The power of these frequencies was much higher in cultures grown on nC films as compared to glass. Addition of blockers of all major sources that could drive influx of extracellular calcium [TTX (1 μM), CNQX (10 μM), APV (50 μM), BMI (20 μM), as well as blockers of L-type voltage-gated calcium channels NiCl_2_ and CdCl_2_ (100 μM each); hereafter referred to as “blockers”] dramatically reduced amplitude of calcium transients (Fig. 4a) and oscillations in the 0–0.02 Hz frequency band (Fig. 4d) as compared to recordings in control ACSF solution.

These data indicate that culturing of neurons on nC films results in a very early increase in intrinsically-evoked calcium oscillations that support a precocious neuronal maturation. This novel mechanism may also drive the trophic effect of the different types of carbon materials.

## Conclusion

In conclusion, we have shown that nC substrate induces augmented oscillations of intracellular calcium in neurons at a very early developmental stage (DIV 1), and premature development, which later on manifests as increased network activity (DIV 3 and DIV 7), up-regulation of KCC2 (DIV 3 and DIV 7), and increased arborisation (DIV 7). The effect appears not to be universal to all carbon materials, as ta-C did not have the same effect. As both carbon substrates are smooth and do not possess notable topographies, a plausible mediator of the trophic effect of these materials could be the differences in the electrical properties, the sp^3^ and sp^2^ hybridizations and the crystallinities of the two carbon materials. At the same time, conductive properties of nC alone are not sufficient to induce precocious maturation of neurons, as an alternative conductive material ITO does not enhance early intrinsic oscillations (Fig. S3c). Overall, nanocarbon material promotes the survival and maturation of neurons, which could be useful for therapeutical applications and implantable devices in the future.

## Supplementary information

Supplementary Figures.
