# Supplementary material for: Novel carbon film induces precocious calcium oscillation to promote neuronal cell maturation
Source: Sci Rep. 2020 Oct 19;10:17661. doi: 10.1038/s41598-020-74535-6 (PMC7573613; doi:10.1038/s41598-020-74535-6)
Supplement: Supplementary file 1 — Supplementary Figures. [file 41598_2020_74535_MOESM1_ESM.pdf]

Novel carbon film induces precocious calcium oscillation to promote neuronal cell maturation

Anastasia Ludwig[1]‡, Sebnem Kesaf[1]‡, Joonas J. Heikkinen[2]‡, Tatiana Sukhanova[1], Shokoufeh Khakipour[1]§, Florence Molinari[5], Christophe Pellegrino[4,5], Sung I. Kim[3], Jeon G. Han[3], Henri J. Huttunen[1], Sari Lauri[1], Sami Franssila[2], Ville Jokinen[2] and Claudio Rivera[1,4,5]**

SUPPLEMENTARY FIGURES

Figure S1. NeuN (green) and KCC2 (red) immunostaining of cells grown on glass and ta-C substrates.

Figure S2. Relative number of active cells in cultures grown on the NC substrate at DIV 3 and DIV 7 (individual values are plotted as open circles, mean values are plotted as horizontal lines, error bars represent SEM), the number of active cells in age-matched cultures grown on glass is set to 1 and marked with the dashed line (mean values, error bars represent SEM; 3 independent cultures; coverslips recorded at DIV 3 n=22; at DIV 7 n=10).

Figure S3. Calcium activity in neurons cultured on ITO substrate: a) Relative number of active cells in cultures grown on the ITO substrate at different DIV (individual values are plotted as open circles, mean values are plotted as horizontal lines, error bars represent SEM), the number of active cells in age-matched cultures grown on glass is set to 1 and marked with the dashed line (mean values, error bars represent SEM; 3 independent cultures; coverslips recorded at DIV 1 n=3; at DIV 3 n=5; at DIV 7 n=6); b) representative traces of intracellular calcium fluctuations at DIV 1 in the presence of TTX

or the blocker cocktail; c) mean power spectral density curves of neuronal activity frequencies at DIV 1 before and after the blockers application (2 independent cultures; TTX n=99, blockers n=8 cells recorded).

S1

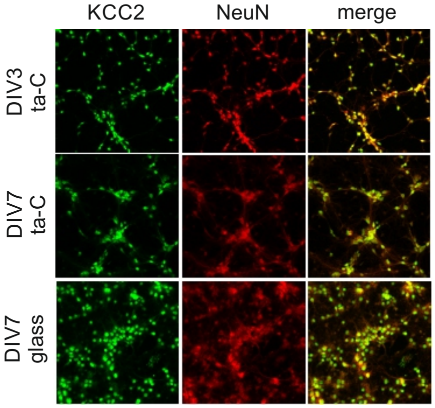
KCC2
NeuN
merge
DIV3 ta-C
DIV7 ta-C
DIV7 glass

S2

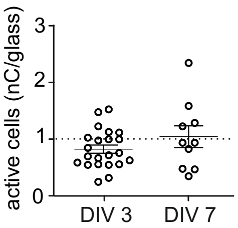
active cells (nC/glass)
0
1
2
3
DIV 3
DIV 7

S3

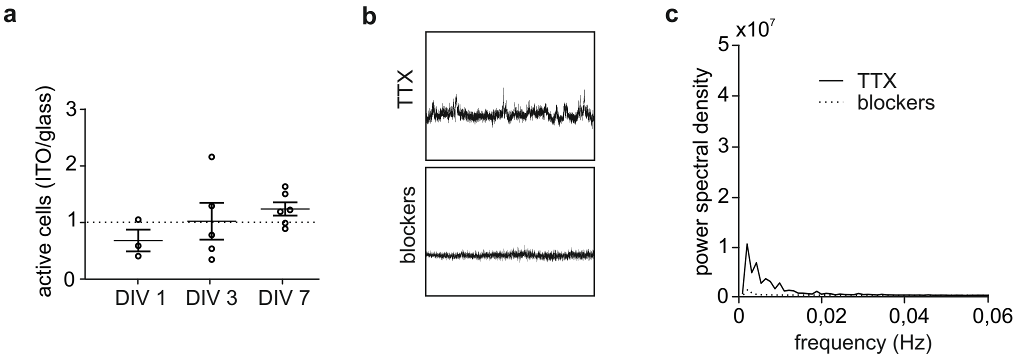
a
active cells (ITO/glass)
0
1
2
3
DIV 1
DIV 3
DIV 7
b
TTX
blockers
c
x10^7
power spectral density
0
1
2
3
4
5
TTX
blockers
0
0,02
0,04
0,06
frequency (Hz)
